# Ultrastructure of a Mobile Threadlike Tissue Floating in a Lymph Vessel

**DOI:** 10.1155/2016/3064072

**Published:** 2016-03-01

**Authors:** Sharon Jiyoon Jung, HyunJi Gil, Dong-Hyun Kim, Hong-Lim Kim, Sungchul Kim, Kwang-Sup Soh

**Affiliations:** ^1^Nano Primo Research Center, Advanced Institute of Convergence Technology, Seoul National University, Suwon 443-270, Republic of Korea; ^2^Department of Transdisciplinary Studies, Graduate School of Convergence Science and Technology, Seoul National University, Suwon 443-270, Republic of Korea; ^3^Integrative Research Support Center, College of Medicine, The Catholic University of Korea, Seoul 137-701, Republic of Korea; ^4^Department of Acupuncture & Moxibustion, Wonkwang University, Gwangju Medical Hospital, Gwangju 503-310, Republic of Korea

## Abstract

Observations of the primo vascular system (PVS) floating in lymph ducts were reported by various groups. There have been, however, no studies on the ultrastructure of the entire cross section of a primo vessel (PV) inside a lymph vessel with a transmission electron microscope (TEM). In the current study we took the TEM images of a cross section of the PV inside a lymph vessel. We used the Alcian blue staining method for the finding of the target PV in a lymphatic vessel by injecting the dye into the inguinal lymph nodes. The stained PV was harvested together with the lymph vessel and some parts of the specimens were used for studying with optical microscopes. Some other parts were treated according to a standard protocol for TEM. As the results the TEM study revealed the loosely distributed collagen fibers with plenty of empty spaces and the lumens with the endothelial nuclei. It turned out to be very similar to the ultrastructure of the PVs observed on the surfaces of internal organs. It also showed how compactly the PV is surrounded with lymphocytes. In conclusion, the detailed morphological features like the distribution of fibers in the PV were revealed and shown to be similar to another kind of the PV on the surfaces of internal organs.

## 1. Introduction

Korea has practiced acupuncture for thousands of years and made many original contributions. For instance, Saam acupuncture was developed by Saam several hundred years ago [1], and the “Constitutional Medicine” by JM Lee was introduced more than a hundred years ago [2]. More recently, in the early 1960s, Kim claimed that his team discovered the anatomical structure of acupuncture meridians as a circulating system which governs the regeneration of damaged and aged tissues and organs [3]. This system has been revived since 2002 and renamed as the primo vascular system (PVS) [4] and its relation to acupuncture is to be established even though relevant data are being accumulated [5].

The medical significance of the PVS besides the relation to acupuncture is steadily increasing. For example, the abundance of mast cells and other innate immune cells in the PVS [6] suggests a major role of the PVS in the innate immune functions. The regeneration function of the PVS that Kim claimed and emphasized [3] was also strongly supported by the discovery of the abundance of embryonic-like stem cells in the PVS [7].

The PVS in which stem cells were found were harvested from the blood vessels and lymph vessels. The primo vessel (PV) afloat in the lymph flow as a mobile threadlike structure is hard to observe without a suitable staining. Most frequently used staining dye was Alcian blue [8], and recently hollow gold nanoparticles were found to be more effective for visualizing purpose [9]. Although numerous articles on the observation of the lymphatic PVS were reported, there has not been a thorough study of the ultrastructure of the PV with transmission electron microscope (TEM). A previous TEM study of a lymphatic PV showed only part of the PV cross section in comparison with a lymph vessel [10]. Therefore the detailed histological characterization of the PV has not been given in comparison with the lymphocytes that surround the PV. In this paper we present the whole view of the cross section of the PV compactly surrounded by lymphocytes so that the characteristic histological difference of the PV from the lymphocytes can be seen despite their similar sizes. Consequently, the current work firmly establishes the existence of the PV by providing firm data showing its characteristic ultrastructure.

## 2. Materials and Methods

### 2.1. Animals

Rats (Sprague-Dawley, male, 7 weeks old, 210~230 g) were obtained from DooYeol Biotech (Seoul, Republic of Korea) and housed in a temperature-controlled environment (23°C). All animals were exposed to a 12-hour light-dark cycle and were provided food and water* ad libitum*. The procedures involving the animals and their care were in full compliance with current international laws and policies (*Guide for the Care and Use of Laboratory Animals*, National Academy Press, 1996) and were approved by the Institutional Ethics Committee of the Advanced Institute of Convergence Technology (Approval number: WJIACUC20150804-3-04). The rats were anesthetized by intramuscular injection of a regimen consisting of 1.5 g/kg of urethane and 20 mg/kg of xylazine.

### 2.2. Visualization and Observation of the Primo Vascular System

Rats were injected with Alcian blue, and the 1.0% Alcian blue (A5268, Sigma-Aldrich, St. Louis, MO, USA) solution in boiled phosphate-buffered saline (PBS, pH 7.4) was filtered by using a 0.22 *μ*m membrane filter (Merck Millipore, Darmstadt, Germany). After the inguinal node had been exposed, the prepared 1.0% Alcian blue dye was injected into the node. The lymph duct from the inguinal node to the axillary node was exposed to observe the PV in it. After the rats were sacrificed by using an intracardiac injection of urethane (1 mL), the lymph duct, including the PV, was harvested for histological study.

To confirm the distribution of rod-shaped nuclei with 4′,6-diamidino-2-phenylindole (DAPI), the specimen was stained with 300 nM DAPI (D1306, Invitrogen, MO, USA) solution for 20 minutes. The shape and distribution of nuclei were examined under a phase contrast microscope (Olympus, U-LH100HG, Japan) and a confocal laser scanning microscope (CLSM; C1 plus, Nikon, Japan).

For the observations with TEM, the specimens of the PV within a lymph vessel were fixed in 4% paraformaldehyde and 2.5% glutaraldehyde in 0.1 M phosphate buffer for overnight. After washing in 0.1 M phosphate buffer, the specimens were postfixed with 1% osmium tetroxide in the same buffer for 1 hr. The specimens were dehydrated with a series of the graded ethyl alcohol, and pure acetone. The specimen was embedded in Epon 812 and the polymerization was performed at 60°C for 3 days. The cross sections of the PV inside a lymph vessel were first studied with optical microscope images of the semithin sections (1 *μ*m) of the specimen before taking ultrathin sections (60–70 nm) for TEM. These semithin section images helped us to find the appropriate location for TEM. Ultrathin sections (60–70 nm) were obtained by ultramicrotome (Leica Ultracut UCT, Germany). Ultrathin sections collected on grids (200 mesh) were examined in TEM (JEM 1010, Japan) operating at 60 kV and images in the TEM were recorded by CCD camera (SC1000, Gatan, USA). Length on the electron micrograph was measured using GMS software (Gatan, USA).

## 3. Results

**Figure 1:**
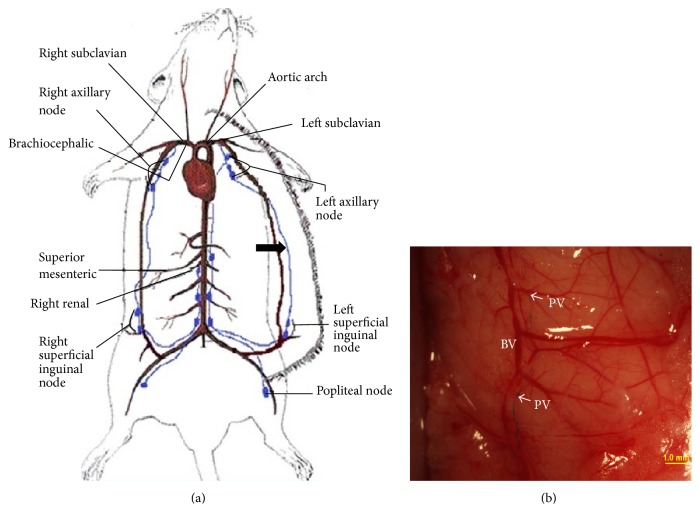
Stereomicroscopic images of lymph ducts in which a PVS was stained with Alcian blue. (a) Illustration of the locations of the lymph nodes and ducts along the epigastric blood vessels (thick arrow) in skin. (b) The blue stained primo vessel (PV) in the lymph duct along the blood vessel (BV) is indicated with arrows. The lymph vessel is hardly visible in a stereomicroscopic image.

The lymph vessel we studied was the one starting from the inguinal node, running along the epigastric blood vessel in the skin and entering the axillary node (Figure 1(a)). The PV in this lymph vessel was observed* in vivo in situ* as a mobile floating threadlike structure stained blue with Alcian blue (Figure 1(b)).

**Figure 2:**
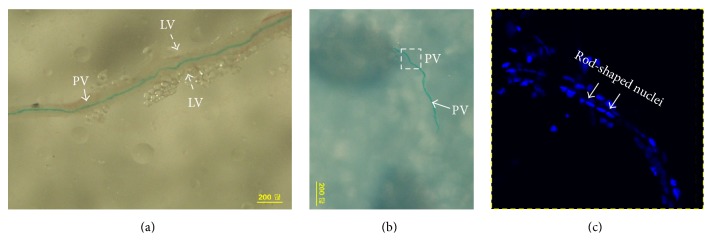
Images of a harvested lymph duct in which a PV was stained with Alcian blue. (a) A piece of the lymph vessel (LV) was harvested and image was taken with a phase contrast microscope. The PV is clearly seen due to the Alcian blue staining. (b) The stained PV was extracted from the lymph vessel with a forceps. It was treated with DAPI to stain its nuclei. (c) The boxed region of the PV in (b) was examined with a confocal microscope. The rod-shaped nuclei of the endothelial cells of the PV were observed at the 15 *μ*m depth from the surface of the stained threadlike structure whose thickness was 23 *μ*m. The PV became thicker because it was covered with lymphocytes.

The lymph vessel with the PV inside was harvested as shown in Figure 2(a). A piece of PV was extracted from the lymph vessel (Figure 2(b)) and stained with DAPI for studying nuclei distribution in the PV. It was examined with a confocal microscope. As shown in the cross section view below the PV was not pure but rather thickly shrouded with lymphocytes; consequently the nuclei seen in the surface of the apparent threadlike structure had round shapes. The optical sections of five *μ*m thickness revealed the characteristic rod-shaped nuclei at the 15 *μ*m depth from the surface of the extracted specimen whose thickness was 23 *μ*m. It means the PV was located somewhat off-centered in the harvested specimen of the stained threadlike structure. The lengths of the rod-shaped nuclei were about 10 *μ*m (Figure 2(c)).

**Figure 3:**
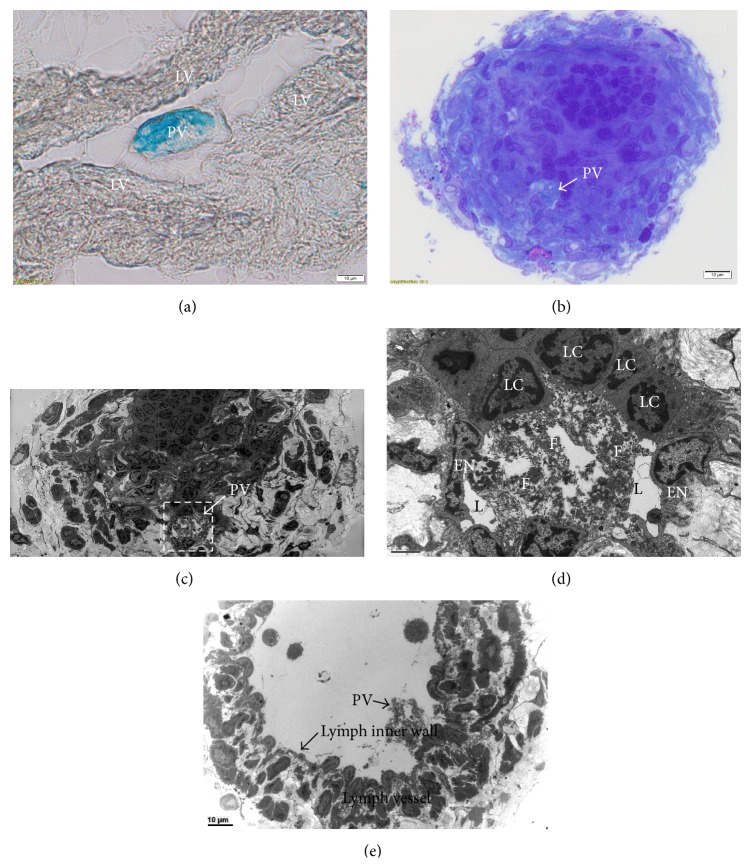
Cross-sectional images of a PV in the harvested lymph duct. (a) A cross section of the lymph duct (LV) with a blue stained PV in it. This specimen was frozen in OCT and the image was taken with a phase contrast microscope. This part of the lymph duct contained the PV without surrounding lymphocytes. (b) The semithin section of the toluidine blue stained lymph duct which were full of lymphocytes. The PV was located below from the center of the lymph duct and it was somewhat light blue colored because of the Alcian blue. This image helped us to find the PV among the lymphocytes. This semithin image is necessary for pointing the precise location of the PV, which is in turn helpful to apply TEM study. (c) The mosaic of the TEM images of the lymph duct which was full of lymphocytes. The location of the PV was identified with the aid of the above toluidine image. The PV showed the loose distribution of collagen fibers. (d) The boxed region of (c) is magnified to show the details of the PV. Two endothelial cell nuclei (EN) are seen along the lumens (L) which are different from the nuclei of the surrounding lymphocytes (LC). The collagen fibers (F) are extracellular material filling most of the PV. (e) A lymph vessel in which a PV was found without surrounding lymphocytes. The PV has similar distribution of fibers and lumens as (d).

A piece of the harvested lymph vessel was embedded in OCT and its micro section was examined after defreezing (Figure 3(a)). The Alcian blue stained threadlike structure containing the PV is clearly isolated from the lymph vessel which looked nearly void of lymphocytes. But another section we took for the TEM study was full of lymphocytes as shown in the toluidine blue stained semithin section (Figure 3(c)). Among the deep dark blue stained lymphocytes in the lymph vessel laid the light blue stained PV. Thanks to the precise positioning of the PV in the semithin section we were able to identify the PV in the TEM images (Figure 3(c)) which is a mosaic of several photographs to cover the whole lymph vessel. The PV is compactly surrounded by lymphocytes and is hard to recognize it unless its location is given by other means. The confounding factors are the small size of the PV and the thinness and softness of the surrounding membrane of the PV which makes the distinction of the PV from the adhered materials.  Figure 3(d) shows the PV with its two endothelial nuclei and loosely scattered distribution of collagen fibers with many empty spaces.

## 4. Discussion

The PV inside lymph vessel has been identified through several stages of examinations: The PV stained blue with Alcian blue* in vivo in situ *is to stay afloat unbroken and not crushed to pieces when the lymph vessel is pushed and moved by a forceps. When the lymph vessel is harvested the PV should be strong enough to be extracted through a hole by pulling one end of the PV with forceps. Most critically, the PV after staining with DAPI should show the presence of the rod-shaped nuclei distributed in broken lines along the direction of the lymph vessel, which can be seen with a confocal microscope. Figures 1(b), 2(a), 2(b), and 2(c) show that the specimen we studied passed these identification criteria.

The main contribution of the present work was to show the ultrastructure of the PV in the lymph vessel with TEM images. Although the TEM images of the lymphatic PV were previously taken they were only small parts of the PV and lymph vessel [10]. This time we took the whole cross-sectional view of the containing lymph vessel and the PV. It showed the characteristics loose distribution of the collagen fiber with many empty spaces, which was in good agreement with the previous work of the lymphatic PV [10] and the PV on the organ surfaces [11]. The thin surrounding membranes of the PV are another agreeing feature which is very thin as Kim described [3]. In addition, it revealed the endothelial nuclei and the lumens as shown in Figures 3(c) and 3(d). This is the most important histological property that could be examined with TEM. This detailed ultrastructural characteristic can only be seen with TEM and is necessary to identify the PV in a complicated situation incurred by the accumulation of lymphocytes. Without the help of the semithin section stained with toluidine blue in Figure 3(b) it would very difficult to locate the position of the PV.

The medical significance of the PVS is still to be investigated but data have already been accumulated showing its possible roles in innate immunity suggested by the abundant immune cells like mast cells in the PVS [6, 11]. There are also data showing the role as a source of very small stem cells [7], a path for metastasis [12], and a haven for cancer stem cells [13].

In conclusion, the study of the ultrastructural morphology of the lymphatic PV showed its detailed structure enough to identify the PV among the complicated situation when compactly surrounded with lymphocytes. This also explains how difficult it is to find the PV without careful and intentional search for it with TEM.
